# Eradication of *Helicobacter pylori* alleviates lipid metabolism deterioration: a large-cohort propensity score-matched analysis

**DOI:** 10.1186/s12944-022-01639-5

**Published:** 2022-04-03

**Authors:** Zeyu Wang, Weijun Wang, Rui Gong, Hailing Yao, Mengke Fan, Junchao Zeng, Sanping Xu, Rong Lin

**Affiliations:** 1 Department of Gastroenterology, Union Hospital, Tongji Medical College, Huazhong University of Science and Technology, Wuhan, 430022 China; 2 Health Management Center, Union Hospital, Tongji Medical College, Huazhong University of Science and Technology, Wuhan, 430022 China

**Keywords:** *Helicobacter pylori*, Eradication treatment, Lipid metabolism, High-density lipoprotein (HDL), Low-density lipoprotein (LDL), Lipid deterioration

## Abstract

**Background:**

The impact of *Helicobacter pylori* (*H. pylori*) eradication on metabolism of lipid and the potential predictor of such changes remain unclear.

**Methods:**

This study retrospectively included subjects who underwent at least two ^13^C urea breath tests between 2015 and 2019 at Wuhan Union Hospital. Based on two *H. pylori* ^13^C examination results, subjects were divided into propensity score-matched persistently negative (HPN), persistently positive (HPP), and eradication (HPE) groups. The changes in lipid measurements from before to after *H. pylori* eradication, including high-density lipoprotein (HDL), low-density lipoprotein (LDL), total cholesterol, and triglycerides, were compared within and between groups. Forty-two candidate factors were tested for their ability to predict lipid metabolism changes after *H. pylori* eradication.

**Results:**

After propensity score matching, 3412 matched cases were analyzed. Within-group comparisons showed significantly decreased HDL (*P* <  0.001) and increased LDL (*P* <  0.001) at the second examination in both the HPE and HPP groups. Between-group comparisons showed that the HDL decrease of the HPE group was significantly larger and smaller when compared with the HPN (*P* = 0.001) and HPP (*P* = 0.004) group, respectively. Uni- and multivariate analyses showed that low diastolic blood pressure (DBP) (*P* = 0.002) and high mean platelet volume (MPV) (*P* = 0.001) before eradication were associated with increased HDL after eradication. Low total protein (TP) (*P* <  0.001) was associated with decreased LDL after eradication.

**Conclusions:**

Compared with sustained *H. pylori* infectious states, *H. pylori* eradication alleviated the lipid metabolism deterioration but did not restore it to the uninfected level within 1.5 years after eradication. Patients with low DBP, high MPV, and low TP may reap a greater lipid-metabolism benefit from *H. pylori* eradication.

## Background

*Helicobacter pylori* (*H. pylori*) is one of the most prevalent infectious factors in the world [1]. *H. pylori* infection can cause a low degree of inflammation in the digestive tract, leading to digestive system diseases such as chronic gastritis, gastric ulcer, and gastric carcinoma [2]. *H. pylori* infection may also mediate distal diseases outside the digestive tract (extra-GI), such as metabolic syndrome [3] and nonalcoholic fatty liver disease [4]. By changing the distribution of plasma lipids [5, 6], lipid metabolism may play a vital role in *H. pylori* inflammation-mediated extra-GI diseases. Chronic *H. pylori* infection can change the lipid distribution by activating proinflammatory factors, stimulating the synthesis of de novo fatty acids in the liver, and affecting lipolysis [7]. Lipid levels can also be affected through direct liver dysfunction, as *H. pylori* increases small intestinal mucosal permeability, allowing bacterial endotoxins to invade the liver through portal vein and cause hepatic tissue damage [8]. *H. pylori* have been found to be an independent risk factor for impaired lipid profiles [9, 10] manifested as reduced high-density lipoprotein (HDL) and elevated low-density lipoprotein (LDL) levels.

Although *H. pylori* infection affects lipid metabolism, whether eradication treatment affects lipid profile is still debated. In a Spanish study, patients who received successful *H. pylori* eradication had better serum HDL than patients with consistent infection [11]. In contrast, other study saw no significantly different LDL, triglyceride (TG) or total cholesterol (TCH) levels between continuously infected and successfully eradicated patients [12]. Notably, these studies enrolled only a relatively small number of patients. A healthy control group, i.e., subjects who were uninfected with *H. pylori*, was unavailable in the above two studies, rendering their results less convincing. Another study published in 2018 compared lipid profiles among patients without *H. pylori* infection and patients with *H. pylori* infection but with or without successful eradication [13]. However, essential influencers of lipid metabolism, such as sex, age, and body mass index (BMI), were not strictly controlled, lowering the comparability between groups. In addition, potential predictors of lipid change after eradication were not explored.

Therefore, in this study, a large cohort of subjects was enrolled and compared by dividing into *H. pylori* eradication, consistently infected, and consistently uninfected group. Propensity score matching (PSM) was conducted to control covariates among groups. This study aimed to explore 1) how *H. pylori* eradication affects lipid metabolism and 2) the potential predictors of lipid metabolism changes after *H. pylori* eradication. The answers to these questions could provide evidence on whether *H. pylori* eradication can benefit lipid metabolism and in which population the benefits can be maximized.

## Method

### Participant identification

The medical examination data of the Medical Examination Center of Wuhan Union Hospital from 2015 to 2019 were retrospectively collected. All cases were collected consecutively according to the inclusion and exclusion criteria.

Cases were included if 1) the *H. pylori* ^13^C urea breath test was conducted; 2) at least two medical examination data points for the *H. pylori* detection were recorded; and 3) lipid metabolism parameters, including TCH, TG, HDL, and LDL, and abdominal ultrasound were recorded in the medical examination data.

Exclusion criteria: 1) existence of abdominal malignant lesions such as liver cancer or structural lesions such as cirrhosis, as indicated by ultrasound; 2) existence of thyroid dysfunction, as indicated by abnormal T3, T4, and thyroid-stimulating hormone levels; 3) existence of a self-reported hepatitis A, B or C history; 4) existence of a self-reported history of abdominal surgery; 5) lack of basic demographic data such as age and sex.

Wuhan Union Hospital approved this study. Informed consent was deemed unnecessary due to prior patient information anonymization.

### Participant grouping and data collection

*H. pylori* infection was diagnosed according to the *H. pylori* ^13^C urea breath test results. Test results were expressed as the delta over baseline (DOB) value, and DOB ≥ 4.0 was defined as positive *H. pylori* infection. Given that all subjects had at least 2 test records, subjects were divided into three groups based on the results of multiple *H. pylori* ^13^C tests: 1) persistently negative group (HPN), where subjects never tested positive; 2) persistently positive group (HPP), where subjects always tested positive; and 3) eradication group (HPE), where subjects tested positive first and then negative. Subjects who tested negative and then positive were not included in the analysis due to their small number (*n* = 137). Data were collected from two examinations for each subject. For subjects in the HPE group, data were collected from the two consecutive examinations where the ^13^C urea breath test turned from positive to negative. For subjects in the HPN and HPP groups, data were collected from the last two examinations. Notably, the last two examinations, rather than the first and the last, were used for analysis so that all three groups would be judged on two consecutive examinations.

For each examination, the following data were systematically collected: 1) lipid metabolism parameters, including TCH, TG, HDL, and LDL; 2) demographic information, including age, sex, height, body weight, BMI, systolic blood pressure (SBP), diastolic blood pressure (DBP) and fasting blood glucose; 3) comorbidities, including abdominal-ultrasound confirmed fatty liver and cholelithiasis; 4) hematological indices, including white blood cell count, neutrophil count, neutrophil percentage, peripheral blood lymphocyte, basophil count, basophil percentage, eosinophil count, eosinophil percentage, red blood cell count, red blood cell volume, mean corpuscular hemoglobin, mean corpuscular hemoglobin concentration, mean corpuscular volume, red blood cell volume distribution width, mean platelet volume, hemoglobin, and platelet count; 5) renal function indices, including creatinine, urine uric acid, urine pH, and blood urea nitrogen; and 6) liver function indices, including albumin, globulin, albumin/globulin, total protein (TP), γ-glutamate transpeptidase, alanine aminotransferase, aspartate aminotransferase, alkaline phosphatase, direct bilirubin and total bilirubin.

### Propensity score matching (PSM)

Given that confounders such as age and sex may significantly influence lipid metabolism parameters and that the HPP and HPN groups were significantly larger than the HPE group, patients in the HPP and HPN groups were 1:1 propensity score-matched (PSM) to patients in the HPE group. Four features, age, sex (which was transformed to a dummy variable with values of 0 and 1), BMI, and the time gap between the two examinations, were utilized to estimate the propensity score using a logistic regression model (Python method *LogisticRegression*). Matching was then performed based on the estimated propensity score employing the nearest neighbor approach (Python method *NearestNeighbors*) with a caliper width of 0.20. Propensity score estimation and matching were done by the Python *sklearn* package (https://scikit-learn.org/stable/).

### Statistical analysis

Continuous variables are presented as mean and standard deviation (SD), and count variables are presented as N and percentage. Within-group comparisons between two time points were conducted using the paired t test. The change value of each of the four lipid measurements between the two examinations was calculated as:

$$ {Change}^k={value}_{post}^k-{value}_{pre}^k $$

where k represents TCH, TG, HDL, or LDL; post represents the second examination, and pre represents the first examination.

One-way analysis of variance (ANOVA) with Tukey’s post hoc analysis was used for between-group comparisons of the change values among the three groups. Further analysis of covariance (ANCOVA) of the change value was performed to adjust for potential confounders, including age, sex, BMI, blood pressure, and hepatic and renal function measurements. To validate the results obtained from the analysis based solely on change value, ANCOVA for the post value were further employed when the pre value was set as a covariate to be adjusted. An estimated marginal mean of the post value was generated and compared between groups.

For the lipid measurements that were significantly different between the two examinations in the HPE group, correlates that were potentially predictive of the change were further explored. This was done by dividing subjects in the HPE group into two subgroups according to the change value between the two examinations: the lipid metabolism increased group (where the change value was > 0) and the lipid metabolism decreased group (where the change value was ≤0). Then, the candidate measurements were compared between the two subgroups using the independent t test for continuous variables and the chi-square test for count variables. In total, 42 candidate measurements were included (as mentioned in participant grouping and data collection). *P* values were corrected to 0.05/42 = 0.0012 by Bonferroni correction. Identified significant factors from the univariate analysis were further eligible for entering a stepwise-multivariate logistic regression model. Statistically significant was set as a 2-tailed *P* <  0.05. Python 3 were used for all statistical analyses.

## Results

### Patient characteristics

**Fig. 1:**
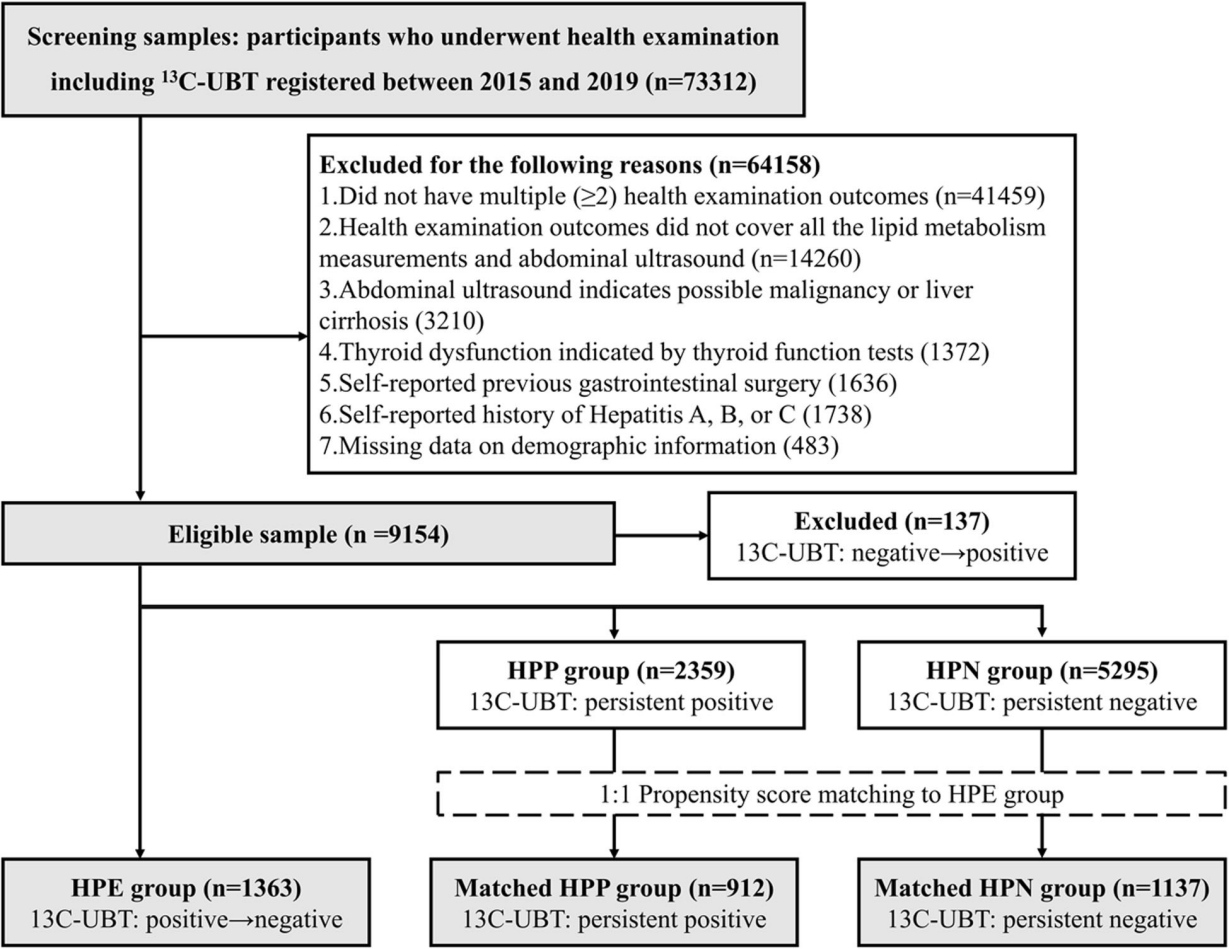
The selection and analytic workflow of the study participants

A total of 73,312 subjects who underwent the *H. pylori* ^13^C urea breath test from 2015 to 2019 of Wuhan Union Hospital Medical Examination Center were identified. According to the inclusion and exclusion criteria, 9017 subjects were included in the analysis before PSM (*n* = 1363 in the HPE group, *n* = 2359 in the HPP group, and *n* = 5295 in the HPN group). After PSM, 912 subjects in the HPP group and 1137 subjects in the HPN group were matched to subjects in the HPE group (*n* = 1363), resulting in a total sample size of 3412 in the three groups. The detailed selection workflow of the study participants is shown in Fig. 1.

The demographic information of the three groups before and after PSM is shown in Table 1. Age, female percentage, BMI, and the time gap between the two examination points among the three groups were no longer significantly different after PSM. For the whole population, the average age, female percentage, BMI, and time gap between the two examinations were 45.3 years, 32.9%, 24.1, and 17.3 months, respectively.

**Table 1:** Baseline characteristics of the HPE, HPP, and HPN groups before and after PSM

	HPE	HPP	HPN	p for ANOVA
Before PSM				
n	1363	2359	5295	
Age (yr)	45.5 ± 12.1	44.8 ± 12.3	43.7 ± 12.7	**< 0.001**
Female percent (n)	33.2% (452)	34.1% (805)	36.7% (1938)	**0.018**
BMI	24.2 ± 3.4	24.2 ± 3.3	23.9 ± 3.3	**< 0.001**
Two-examination time gap (month)	17.1 ± 8.4	19.6 ± 10.0	19.2 ± 10.3	**< 0.001**
After PSM				
n	1363	912	1137	
Age	45.5 ± 12.1	45.3 ± 12.8	45.0 ± 12.6	0.530
Female percent (n)	33.2% (452)	31.9% (306)	33.5% (363)	0.703
BMI	24.2 ± 3.4	24.1 ± 3.2	24.0 ± 3.2	0.472
Two-examination time gap (month)	17.1 ± 8.4	17.3 ± 9.1	17.5 ± 9.0	0.143

*BMI* body mass index, *PSM* propensity score matching

### Within-group comparisons of lipid measurement changes

The comparisons of TCH, TG, HDL, and LDL levels between the two examination points in the three groups are shown in Table 2. In the HPE group, LDL (t = 4.492, *P* < 0.001) and TG (t = 2.699, *P* = 0.007) significantly increased, while HDL (t = 5.072, *P* < 0.001) significantly reduced after eradication. In the HPP group, LDL was significantly increased (t = 6.076, *P* < 0.001), and HDL was significantly decreased (t = 9.228, *P* < 0.001) at the second examination. No significant lipid metabolism change in the HPN group was observed.

**Table 2:** Comparisons of TCH, TG, HDL, and LDL between the 2 examinations in the 3 groups of HPE, HPP, and HPN

	HPE (*n* = 1363)	HPP (*n* = 912)	HPN (*n* = 1137)
TCH first examination	4.80 ± 0.91	4.78 ± 0.87	4.81 ± 0.88
TCH second examination	4.80 ± 0.93	4.75 ± 0.86	4.79 ± 0.91
Change value Δ_TCH_	− 0.01 ± 0.68	−0.03 ± 0.61	−0.02 ± 0.78
Significance for Δ_TCH_	t = 0.165, *p* = 0.868	t = 1.385, *p* = 0.166	t = 1.020, *p* = 0.307
TG first examination	1.58 ± 1.19	1.70 ± 1.45	1.69 ± 1.40
TG second examination	1.67 ± 1.53	1.73 ± 1.61	1.69 ± 1.33
Change value Δ_TG_	0.07 ± 1.04	0.03 ± 1.15	0.00 ± 1.37
Significance for Δ_TG_	**t = 2.699,** ***p*** **= 0.007**	t = 0.804, *p* = 0.422	t = 0.049, *p* = 0.961
HDL first examination	1.39 ± 0.33	1.42 ± 0.35	1.38 ± 0.32
HDL second examination	1.36 ± 0.35	1.35 ± 0.34	1.38 ± 0.34
Change value Δ_HDL_	−0.03 ± 0.23	−0.07 ± 0.22	−0.00 ± 0.25
Significance for Δ_HDL_	**t = 5.072,** ***p*** **< 0.001**	**t = 9.228,** ***p*** **< 0.001**	t = 0.224, *p* = 0.823
LDL first examination	2.78 ± 0.78	2.73 ± 0.76	2.79 ± 0.74
LDL second examination	2.85 ± 0.76	2.83 ± 0.77	2.81 ± 0.74
Change value Δ_LDL_	0.07 ± 0.58	0.11 ± 0.54	0.03 ± 0.64
Significance for Δ_LDL_	**t = 4.492,** ***p*** **< 0.001**	**t = 6.076,** ***p*** **< 0.001**	t = 1.585, *p* = 0.113

*TCH* total cholesterol, *TG* triglycerides, *HDL* high density lipoprotein cholesterol, *LDL* low density lipoprotein cholesterol

### Between-group comparisons of lipid measurement changes

Using one-way ANOVA, the change values of TCH, HDL, LDL, and TG between the two examinations among the three groups were compared. LDL (*P* = 0.009) and HDL (*P* < 0.001) change values varied between groups. A further ANCOVA adjusting for potential covariates was conducted where group (i.e., HPE, HPP, and HPN) was set as an influential factor for HDL and LDL changes. After adjusting for the effects of age, sex, BMI, blood pressure, and hepatic and renal function measurements, the results indicated that group was still a significantly influential factor on HDL and LDL change (Table 3).

**Table 3:** ANCOVA for grouping as an influential factor for HDL and LDL change value Δ

	Grouping as an influential factor for HDL change value	Grouping as an influential factor for LDL change value
Unadjusted	**F = 20.72,** ***p*** **< 0.001**	**F = 4.79,** ***p*** **= 0.009**
Adjusted for age, sex, and BMI	**F = 20.90,** ***p*** **< 0.001**	**F = 4.60,** ***p*** **= 0.010**
Further adjusted for SBP, and DBP	**F = 20.55,** ***p*** **< 0.001**	**F = 4.73,** ***p*** **= 0.008**
Further adjusted for FBG, Scr and BUN	**F = 20.23,** ***p*** **< 0.001**	**F = 4.84,** ***p*** **= 0.008**
Further adjusted for ALT, AST, and GGT	**F = 19.89,** ***p*** **< 0.001**	**F = 4.89,** ***p*** **= 0.007**

*ANCOVA* analysis of covariance, *HDL* high-density lipoprotein cholesterol, *LDL* low-density lipoprotein cholesterol, *BMI* body mass index, *SBP* systolic blood pressure, *DBP* diastolic blood pressure, *FBG* fasting blood glucose, *Scr* creatinine, *BUN* blood urea nitrogen, *ALT* alanine aminotransferase, *AST* aspartate aminotransferase, *GGT* γ-glutamate transpeptidase

**Fig. 2:**
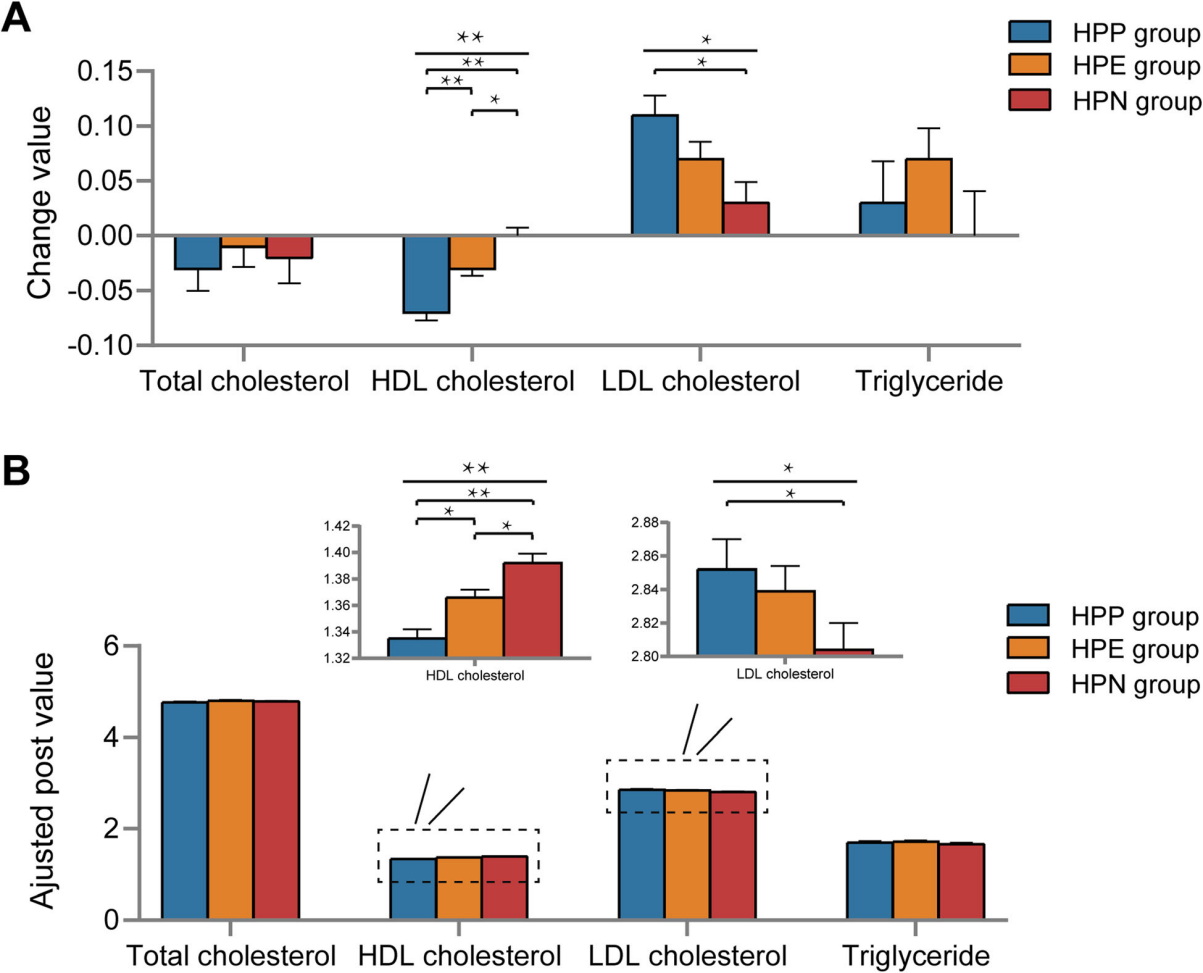
Changes in lipid metabolism parameters between the 2 examination points in the HPP, HPE, and HPN groups. **A**. Bar plots comparing the change values (calculated as the difference between the 2 examinations) among the 3 groups. The error bar represents the standard deviation. **B**. Bar plots showing comparisons of adjusted post values among the 3 groups. The adjusted post value was presented as the estimated marginal means of the lipid metabolism parameters in the second examination adjusted for the first examination’s outcomes. The error bar represents the standard deviation. HPP, persistently positive *H. pylori*; HPE, *H. pylori* eradication; HPN, persistently negative *H. pylori*; HDL, high-density lipoprotein cholesterol; LDL, low-density lipoprotein cholesterol

In the post hoc analysis of one-way ANOVA, HDL was reduced the most, moderately and the least in the HPP, HPE, and HPN group, respectively (*P* for HPP vs. HPN = 0.001, *P* for HPP vs. HPE = 0.004, *P* for HPE vs. HPN = 0.001) (Fig. 2A). LDL increased the most, moderately, and the least in the HPP, HPE, and HPN group, respectively. Significance was observed only between the HPP and HPN groups (*P* = 0.006). No difference in the TCH or TG change value were observed in the three groups.

For the analysis based on change value (Δ), an ANCOVA was conducted on the post value where the pre value was set as a covariate to be adjusted (Fig. 2B). Similar results were obtained: The adjusted post values of LDL (F = 3.381, *P* = 0.034) and HDL (F = 16.690, *P* < 0.001) changed among groups. Post hoc tests showed that the adjusted post value of HDL was lowest, moderate, and highest in the HPP, HPE, and HPN group, respectively (*P* for HPP vs. HPN < 0.001, *P* for HPP vs. HPE = 0.003, *P* for HPE vs. HPN = 0.011). The adjusted post value of LDL was lowest in the HPN group and highest in the HPP group (*P* for HPP vs. HPN = 0.040).

### Exploring correlates of lipid metabolism changes in the HPE group

**Fig. 3:**
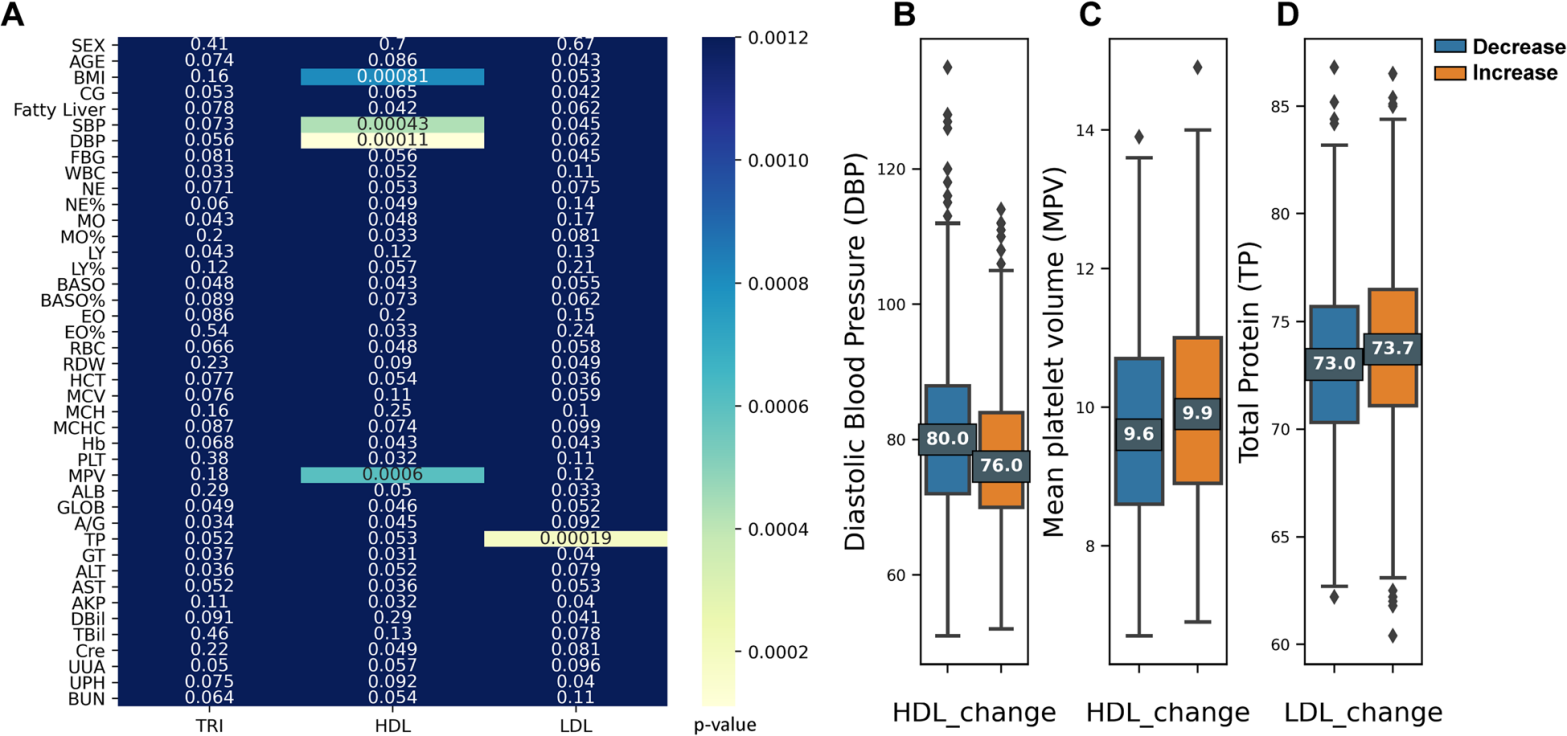
Correlates of lipid metabolism change after *H. pylori* eradication. **A**. Heatmap showing the correlates of TG, HDL, and LDL changes after *H. pylori* eradication. The X-axis shows the 3 lipid metabolism parameters. The Y-axis lists 42 candidate predictors. Different color in each block demonstrates the corresponding *P* value of the comparisons between the increased and the decreased lipid metabolism patients for each candidate predictor by the independent t test. Smaller *P* values are presented by lighter color. *P* < 0.0012 (0.05/42) was determined significant after Bonferroni correction. **B**. Box plots comparing diastolic blood pressure after *H. pylori* eradication between the HDL-decreased and -increased groups. **C**. Box plots comparing mean platelet volume after *H. pylori* eradication between the HDL-decreased and -increased group. **D**. Box plots comparing of total protein after *H. pylori* eradication between the LDL-decreased and -increased group. The upper and lower whiskers on these boxes represent maximum and minimum values, respectively. Each box defines the first and third quartiles, while the marked center value within the box represents the median value. TG, triglycerides; LDL, low-density lipoprotein cholesterol; BMI, body mass index; HDL, high-density lipoprotein cholesterol; CG, calculus of gallbladder; FBG, fasting blood glucose; DBP, diastolic blood pressure; SBP, systolic blood pressure; WBC, white blood cells; NE, neutrophil count; NE%, neutrophil percentage; MO%, monocyte percentage; MO, monocyte count; LY%, lymphocyte percentage; LY, lymphocyte count; BASO%, basophil percentage; BASO, basophil count; EO%, eosinophil percentage; EO, eosinophil count; RBC, red blood cells; RDW, red blood cell volume distribution width; HCT, red blood cell volume; MCHC, mean corpuscular hemoglobin concentration; MCH, mean corpuscular hemoglobin; MCV, mean corpuscular volume; PLT, platelet count; Hb, hemoglobin; MPV, mean platelet volume; ALB, albumin; GLOB, globulin; A/G, albumin/globulin; TP, total protein; GT, γ-glutamate transpeptidase; AST, aspartate aminotransferase; ALT, alanine aminotransferase; AKP, alkaline phosphatase; Cre, creatinine; DBil, direct bilirubin; TBil, total bilirubin; UUA, urine uric acid; UPH, urine pH; BUN, blood urea nitrogen

For the indices (i.e., TG, HDL, and LDL) that significantly changed after *H. pylori* eradication, potential correlates of their changes were explored. Forty-two candidate examination measurements were compared between the lipid-level decrease group and lipid-level increase group, employing the independent t test or chi-square test. The obtained statistics are summarized in a heatmap (Fig. 3A). Significant comparisons after correction indicated zero correlates of the TG change; four potential correlates of the HDL change (BMI (*P* < 0.001), SBP (*P* < 0.001), DBP (*P* < 0.001), and mean platelet volume (MPV) (*P* < 0.001); and one correlate of the LDL change (TP (*P* < 0.001)).

Putting the four factors identified from the univariate analysis into the multivariate logistic regression model employing the enter method, the results showed that BMI (*P* = 0.297) and SBP (*P* = 0.973) were not significant predictors of the HDL change anymore. High DBP (OR = 1.023, 95% CI = 1.008–1.039, *P* = 0.002) and low MPV (OR = 0.877, 95% CI = 0.812–0.948, *P* = 0.001) remained significantly associated with decreased HDL after eradication (Fig. 3B-C). Validations employing forward and backward approaches to build regression models obtained the same outcomes. Since high TP was the only factor associated with increased LDL (Fig. 3D), no multivariate model was needed.

## Discussion

This study retrospectively analyzed the recorded data of subjects with multiple *H. pylori* ^13^C urea breath tests and compared the lipid metabolism profiles among subjects with persistent *H. pylori* infection, subjects with *H. pylori* eradication, and subjects never had a positive breath test. The results showed that HDL and LDL levels continued to deteriorate in the HPE group after eradication. In the HPE group, the HDL deterioration turned more prominent when compared with the HPN group and turned less when compared with the HPP group. This indicated that *H. pylori* eradication may alleviate lipid metabolism deterioration but not restore it to the uninfected level. Regression analysis showed that patients with low DBP, high MPV, and low TP might benefit more from *H. pylori* eradication in terms of lipid metabolism improvement.

After PSM, the average gap between the two physical examination records was approximately 1.5 years. Within the observation window, the HDL and LDL levels were deteriorated among *H. pylori*-eradicated subjects, yet less deteriorated than in continuously positive subjects. The two lipoproteins are closely related to human nutritional metabolism [14–17], are related to arteriosclerosis [18], and are independent predictors of coronary artery disease [19]. The HDL and LDL levels can be altered under *H. pylori* infection through ghrelin and leptin secretion or nutrient absorption imbalance [20–22]. *H. pylori* infection can cause changes in lipid metabolism from acute phase reactions to chronic interleukin release [3, 23, 24], leading to atherosclerotic lipid profiles and elevated cardiovascular risk [25]. According to the results, *H. pylori* infection exacerbated the lipid profile, and eradication partly reversed the exacerbation. Therefore, given its positive effect on controlling and improving the lipid profile, *H. pylori* eradication could be a meaningful way to maintain normal lipid metabolism.

Regression analysis and multivariate analysis showed that high DBP and high TP were risk factors for HDL decrease and LDL increase, suggesting the deterioration of lipoprotein metabolism. HDL is believed to prevent endothelial dysfunction, reduce proinflammatory cell activation, and promote reverse cholesterol transport [26], while LDL is a risk factor for atherosclerosis and is responsible for embolism formation and artery wall retention [27]. *H. pylori* infection can damage the liver [28], where these two lipoproteins are synthesized, secreted, and cleared [29, 30]. Previous cohort studies confirmed *H. pylori* to be an independent predictor of nonalcoholic fatty liver disease [31, 32]. Positive *H. pylori* serum antibodies are more frequently seen in hepatocellular carcinoma patients compared with healthy controls [33]. On the one hand, studies have connected impaired liver function with hypertension through elevated liver enzymes, fat deposition in the liver [34, 35], and secretion of hepatotoxic proinflammatory factors [36]. On the other hand, the elevation of clinical TP could also result in hepatocyte damage or even liver dysfunction [37]. Therefore, considering the influence of high blood pressure and elevated TP on liver function, *H. pylori* eradication would be more beneficial in patients with relatively low DBP and low TP.

The comparison of MPV change values between before and after eradication revealed a less deteriorated lipid profile in the high-MPV population. As one indicator in clinical blood tests, MPV indirectly reflects platelet number and the platelet-producing ability of bone marrow. A previous study suggested that *H. pylori* infection causes platelet destruction and results in an elevated MPV [38]. In contrast, investigations by *Güçlü* et al. [39] and *Topal* et al. [40] found no differences in MPV between *H. pylori*-positive and -negative populations. However, by comparing the effects of eradication with those of both disease control and healthy control status in a large cohort, their results revealed high MPV to be a protective factor of the lipid profile. Like that study, a single-blinded randomized controlled study found MPV counts in *H. pylori* infected people significantly lower when compared to *H. pylori* uninfected people [32]. Therefore, *H. pylori* eradication might bring more ameliorative effect on lipid metabolism in high-MPV patients, and more prospective studies could help flesh out the theory.

### Study strengths and limitations

In this study, a large cohort of patients in the medical center for follow-up visits were included, and their lipid metabolism levels were statistically analyzed. The results confirmed the improvement of lipid metabolism by *H. pylori* eradication and further defined the population who may receive more apparent improvements, which can help with clinical prediction and decision-making.

This study still owed some limitations. First of all, since this analysis retrospectively analyzed medical examination data, the diversity of eradication therapies could not be well controlled, which may introduce potential heterogeneities among individuals. However, published reports have indicated that different eradication therapies of *H. pylori* infection per se did not result in significantly different lipid profiles at the one-year follow-up [41]. Therefore, although this retrospective study could not specify which eradication therapy was used, the change in lipid metabolism caused by eradication should not be affected. Second, the usage of drugs in patients’ daily routine was not known, which may have influenced the monitored indicators. Third, the study retrospectively covered a long time period, so it may not have excluded other confounding factors affecting lipid metabolism, such as lifestyle and eating habit changes. However, comparisons showed no difference in lipid profiles between the two examination points in the HPN group. These results indicate that other factors were unlikely to significantly influence group-level lipid metabolism results, as their effects may have been counterbalanced among individuals, especially when such a large cohort was analyzed. Fourth, the study collected single-center data and was limited to Chinese patients. However, the prevalence of dyslipidemia in this study is consistent with that of other large contemporary trials and real-world registries in non-Chinese populations [42, 43], suggesting the potential generalizability of these results. Last, the study did not include all lipid-related parameters in the analysis. Since clinical data were collected from the medical center where routine health monitoring was conducted, other atherogenic-related lipoproteins, such as lipoprotein (a) [44], were not recorded and therefore were not analyzed here.

## Conclusions

Compared with sustained *H. pylori* infectious states, *H. pylori* eradication alleviated lipid metabolism deterioration but did not restore it to an uninfected level within 1.5 years. Patients with low DBP, high MPV, and low TP may benefit more from *H. pylori* eradication in terms of lipid metabolism. These results provide evidence on whether *H. pylori* eradication benefits lipid metabolism and in which population the benefits can be minimized/maximized. For populations that are highly likely to restore lipid metabolism profiles after *H. pylori* eradication, urgent actions may be suggested by the clinicians to intervene the lipid deterioration of the patients.

## Data Availability

On reasonable request and under permission of Wuhan Union Hospital, data and materials of this study are available from the corresponding author once published.

## Abbreviations

*H. pylori*: *Helicobacter pylori*
extra-GI: Extragastrointestinal
TCH: Total cholesterol
TG: Triglycerides
HDL: High-density lipoprotein
LDL: Low-density lipoprotein
BMI: Body mass index
PSM: Propensity score matching
DOB: Delta over baseline
TP: Total protein
SD: Standard deviation
ANOVA: Analysis of variance
ANCOVA: Analysis of covariance
MPV: Mean platelet volume
DBP: Diastolic blood pressure
SBP: Systolic blood pressure
